# Generic surgical process model for minimally invasive liver treatment methods

**DOI:** 10.1038/s41598-022-19891-1

**Published:** 2022-10-06

**Authors:** Maryam Gholinejad, Egidius Pelanis, Davit Aghayan, Åsmund Avdem Fretland, Bjørn Edwin, Turkan Terkivatan, Ole Jakob Elle, Arjo J. Loeve, Jenny Dankelman

**Affiliations:** 1 Department of Biomechanical Engineering, Faculty of Mechanical, Maritime and Materials Engineering, Delft University of Technology, Delft, The Netherlands; 2 The Intervention Centre, Oslo University Hospital, Oslo, Norway; 3 Institute of Clinical Medicine, Medical Faculty, University of Oslo, Oslo, Norway; 4 Department of Surgery N1, Yerevan State Medical University After M. Heratsi, Yerevan, Armenia; 5 Department of HPB Surgery, Oslo University Hospital, Oslo, Norway; 6 Department of Surgery, Division of HPB and Transplant Surgery, Erasmus MC, University Medical Centre Rotterdam, Rotterdam, The Netherlands

**Keywords:** Medical research, Oncology, Surgery, Therapeutic endoscopy, Therapeutics, Biomedical engineering

## Abstract

Surgical process modelling is an innovative approach that aims to simplify the challenges involved in improving surgeries through quantitative analysis of a well-established model of surgical activities. In this paper, surgical process model strategies are applied for the analysis of different Minimally Invasive Liver Treatments (MILTs), including ablation and surgical resection of the liver lesions. Moreover, a generic surgical process model for these differences in MILTs is introduced. The generic surgical process model was established at three different granularity levels. The generic process model, encompassing thirteen phases, was verified against videos of MILT procedures and interviews with surgeons. The established model covers all the surgical and interventional activities and the connections between them and provides a foundation for extensive quantitative analysis and simulations of MILT procedures for improving computer-assisted surgery systems, surgeon training and evaluation, surgeon guidance and planning systems and evaluation of new technologies.

## Introduction

For many years, surgery has been considered an art, treating surgery as an artist-driven process. This agrees with the fact that many of the processes during surgery are processed mentally inside the artist’s/surgeon’s brain. To better expose this process, expert consensus meetings, national and international guidelines provide generalized recommendations on a high abstraction level based on the pillars of evidence-based medicine. In recent years, with the introduction of new technologies, tools and hybrid operating rooms (ORs), surgeries became increasingly convoluted^1^. Improving these highly complex surgical procedures is a shared concern of experts with different backgrounds. However, without a solid knowledge of these treatment processes, they can hardly be improved^2^.

In surgical process modelling, surgeries are treated not as an artist-driven process but as a sequence of tasks and steps that are followed by the clinical team^3^, which can support analysis and predicting surgical actions. Analysis of surgical process models can reveal the bottlenecks and potential improvements to the surgeries, aiding further advances^4–9^. Such process models are a great means for finding the structural coherence of complex surgical procedures and for obtaining a profound qualitative and quantitative understanding of the relations within the surgical procedure, its variation parameters and its output parameters^10–13^. Hence, these are great tools for training surgical teams and educating young surgeons.

Minimally Invasive Liver Treatment (MILT) is an example of a procedure were different clinicians use different methods and techniques to treat liver lesions through surgical/interventional liver manipulations when non-surgical methods (non-invasive and chemotherapy treatments) are not adequate. After the introduction in the previous century, minimally invasive approach for liver surgery has only in recent years changed the way how benign and malignant lesions are treated^14,15^. Although the less invasive nature of MILT compared to open surgeries benefits the patient^16,17^, various challenges that can increase the risk of surgical errors remain, including inadequate visualization of the patient’s internal structure, lack of tactile feedback and complex navigation towards target treatment lesions^18,19^. Moreover, the continuous change of the liver shape and location due to, e.g., pneumoperitoneum, patient respiration and manipulation of the liver during an intervention, add to these challenges^1^. Over the last three decades, a broad range of MILT techniques has been introduced. These techniques can be categorized into three methods: laparoscopic liver resection (LLR)^20–24^, laparoscopic liver ablation (LLA)^25–29^ and percutaneous ablation (PA)^30–34^ and robot-assisted resection^35^. This paper focuses on the first three categories. As a result, different surgeons and interventionists use different methods and techniques, which can all be executed with large process variations. Furthermore, procedures are further dependent on factors such as:

- medical team skills, experience and preferences
- patient-specific properties, such as patient’s body topography, patient health condition and clinical history
- type, size, and location of the treatment areas.

These all add to the inherent complexity of MILT procedures. A detailed generic process model of MILT is crucial for assessing these complexities, educating new surgeons and improving MILT procedures. Yet, to the best of our knowledge, such model has not yet been established. The sole study available on modeling the MILT process is a qualitative study describing radiofrequency percutaneous ablation^36^. Therefore, the aim of this study is to establish a generic surgical process model (or surgical workflow) of MILT that covers the entire procedure for a variety of MILT methods and their corresponding techniques. The proposed generic process model provides the relation between entities and allows quantitative and qualitative studies of surgical procedure. The process model was developed in a modular way to increase its usability and efficiency and to facilitate aspects of data acquisition, analysis and procedure improvement^10,37–39^.

## Methods

This study focuses on three commonly distinguished MILT *methods*. Within each method several variations, referred to as *types*, can be distinguished:

- **Laparoscopic Liver Resection (LLR):** Resecting the necessary region of the liver parenchyma using the minimally invasive approach. Depending on the size and location of the resection region, three *types* of operations can be applied: formal resection^40,41^, anatomical resection^42–44^ and atypical resection, also known as parenchyma sparing^45–47^.
- **Laparoscopic Liver Ablation (LLA**): Laparoscopic ablation of the tumor by placing one or several needles inside or around the target lesion, aiming to destroy target cells by means of burning, electrifying, freezing, or chemicals. The clinician manipulates the internal structures through small incisions to make the treatment region accessible and to ensure that the treatment is performed on the right location. LLA has four different *types*: Radiofrequency Ablation (RFA)^32–34,48^, Microwave Ablation (MWA)^48–51^, Irreversible Electroporation (IRE)^52^, Cryoablation (CA)^53–55^ and Ethanol Injection (EI)^56–58^.
- **Percutaneous Ablation (PA):** Similar to LLA, but without laparoscopic manipulations and ablation needles are inserted directly through the skin into the treatment area. PA has the same treatment *types* as LLA.

### Modeling strategies

To establish a generic process model of MILT, the modeling strategies proposed in our previous work^10^ were applied as described below.

#### Granularity level

**Figure 1:**
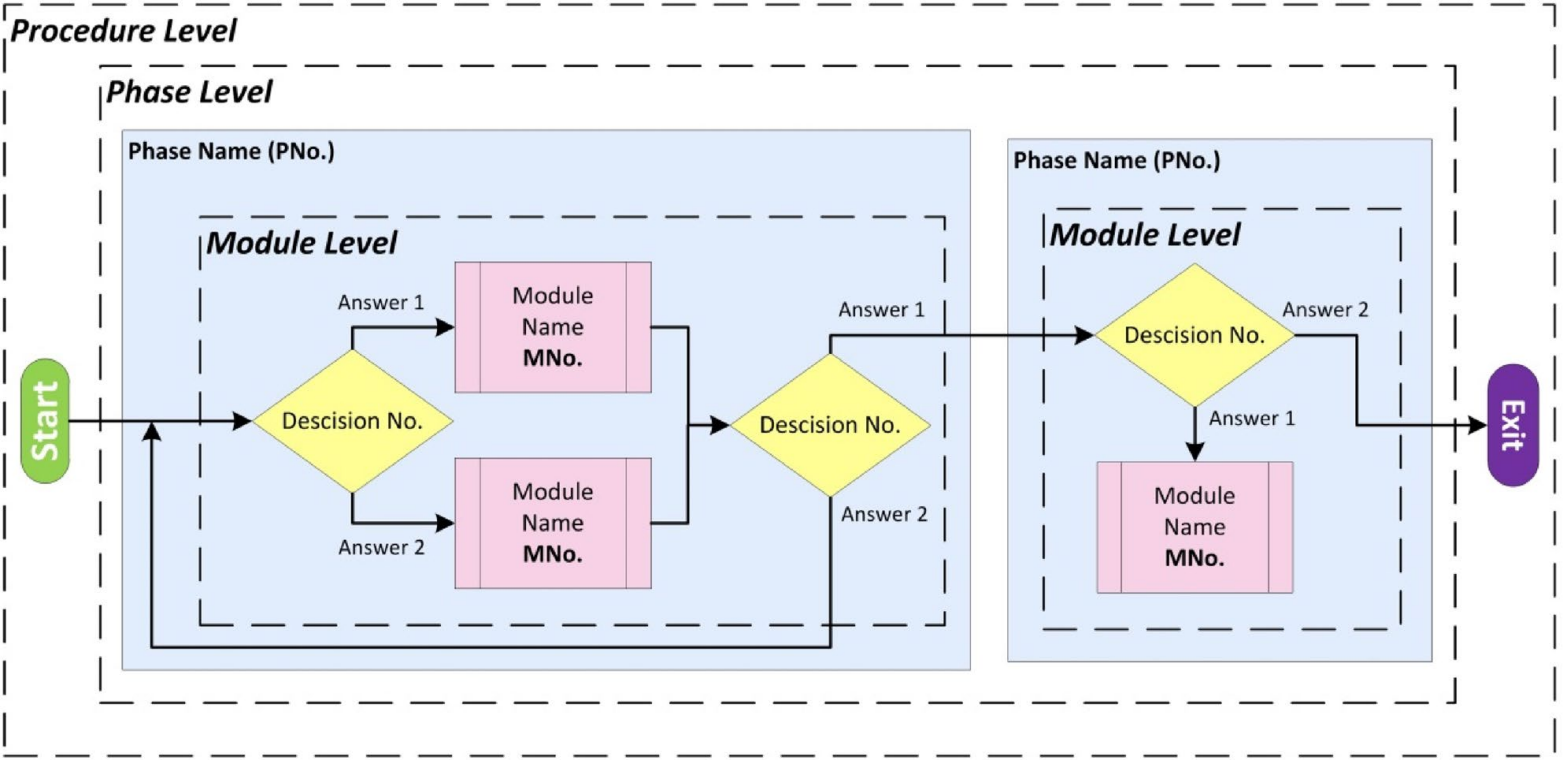
Different levels of granularity embodied in the developed surgical process model.

The generic process model of MILT was established at three levels of abstraction and granularity, see Fig. 1:

- *Procedure* Considering the entire procedure as a single process, starting from patient intake until the end of the intervention. Highest abstraction level, lowest granularity.
- *Phase (P)* Contains groups of modules and decisions that all share a goal or purpose. Intermediate abstraction level, intermediate granularity.
- *Module (M)* A chain of actions and decisions aiming to fulfil a specific goal within a phase. Low abstraction level, high granularity.

#### Data acquisition method

##### Model design data

Data of the MILT procedures were collected through live observations and offline video observations, literature study and interviews.

The data were acquired from:

- Sixteen live observations at Oslo University Hospital (OUH), Oslo, Norway and Erasmus Medical Center, Rotterdam, Netherlands (Erasmus MC), performed by experienced teams. The live observations were composed of twelve laparoscopic and four ablation treatments.
- Eight interviews with clinical experts at Erasmus MC and OUH.
- Nine offline observations using endoscopic video recordings of laparoscopic liver surgeries and OR recordings of ablation procedures.

The process model was primarily designed based on the live observations in the OR. Interviews with the surgical team members were conducted to verify that the observed procedures were representative for MILT methods in general. To obtain a thorough understanding of surgical methods and to let the teams get used to the observer, the observer also attended several laparoscopic resection procedures of other organs in the aforementioned hospitals. Furthermore, the procedure description of MILT procedures in Refs.^19,24,34,40,56,58–65^ has been investigated.

##### Model verification data

After establishing the MILT process model, endoscopic video recordings of laparoscopic liver surgeries of fifteen extra procedures were used for verification. In addition, the author (MG) has attended six intervention sessions in Erasmus MC and Bern University Hospital (BUH).

For final verification, the proposed process model the process model was presented to clinicians and the validity and correctness of the generic process model for different techniques of performing MILT were discussed with the participating clinicians in OUH and Erasmus MC. Example surgical videos were used to discuss how the process model mimics every activities in performing different technics of MILT in clinical practice. Video Marker Software was used to discuss the registered surgical data for the entire duration of sample surgeries over the endoscopic videos.

Ethical approval was obtained from each of the clinical centers in which the data were collected and observations were done for design and verification of the process model (OUH: Regional Ethical Committee of South Eastern Norway- REK Sør-Øst B 2011/1285 and the Data Protection Officer of OUH) and Erasmus MC and BUH with following the hospitals ethical rules). Based on these hospitals rules, informed consents were obtained from patients for further investigation on their surgical procedure. All methods for data acquisition and verification were performed in accordance with the relevant guidelines and regulations of the hospitals.

#### Modelling approach

A combination of top-down and bottom-up approaches was used to benefit from the advantages of both approaches (see^10^). Based on the data from OR observations, interviews and literature studies, a top-down approach was first used to establish a global overview of the surgical workflow. Next, the endoscopic videos were used as low-level data to model the details of the process model and to improve the initially established general overview bottom-up. This modelling process was iterated until no process model changes resulted from new iterations anymore.

#### Generalization

Generalization of the MILT process model to LLR, LLA, PA and their different types and techniques should ensure agreement with divergences and differences of the MILT procedures in clinical practice. Therefore, the data for analysis and modelling was acquired in procedures using various MILT types and techniques, with a variety of patient conditions (age, gender, build, clinical history, tumour specification and number, etc.). The individual procedures were merged in the generalization process, covering all events of the treatments and not only the most probable events.

#### Model representation

The generic MILT process model was concretized by using workflow and process model diagrams. The process model was made to have a modular structure to increase model usability and efficiency^10^.

### Verification

Qualitative and quantitative verifications were done to confirm that the proposed generic process model of MILT reflects the procedure in clinical practice:

- **Qualitative verification** was performed to confirm that the path options in the established process model fit any observed order of possible actions and decisions occurring during MILT in clinical practice. This was done by registering the sequence of the encountered process model elements (phases and modules) throughout the entire treatment procedure of fifteen offline observation of MILT procedures from OUH. In addition, the author (MG) has attended four intervention sessions at Bern University Hospital and two at Erasmus MC. Furthermore, interviews with clinical teams were done and the process model was discussed with highly experienced surgeons with at least 10 years of surgical experience in OUH and Erasmus MC.
- **Quantitative verification** was performed to confirm that the sum of the encountered workflow elements (phases, modules) duration was equivalent to the total procedure times of fifteen offline MILT procedures from OUH.

**Figure 2:**
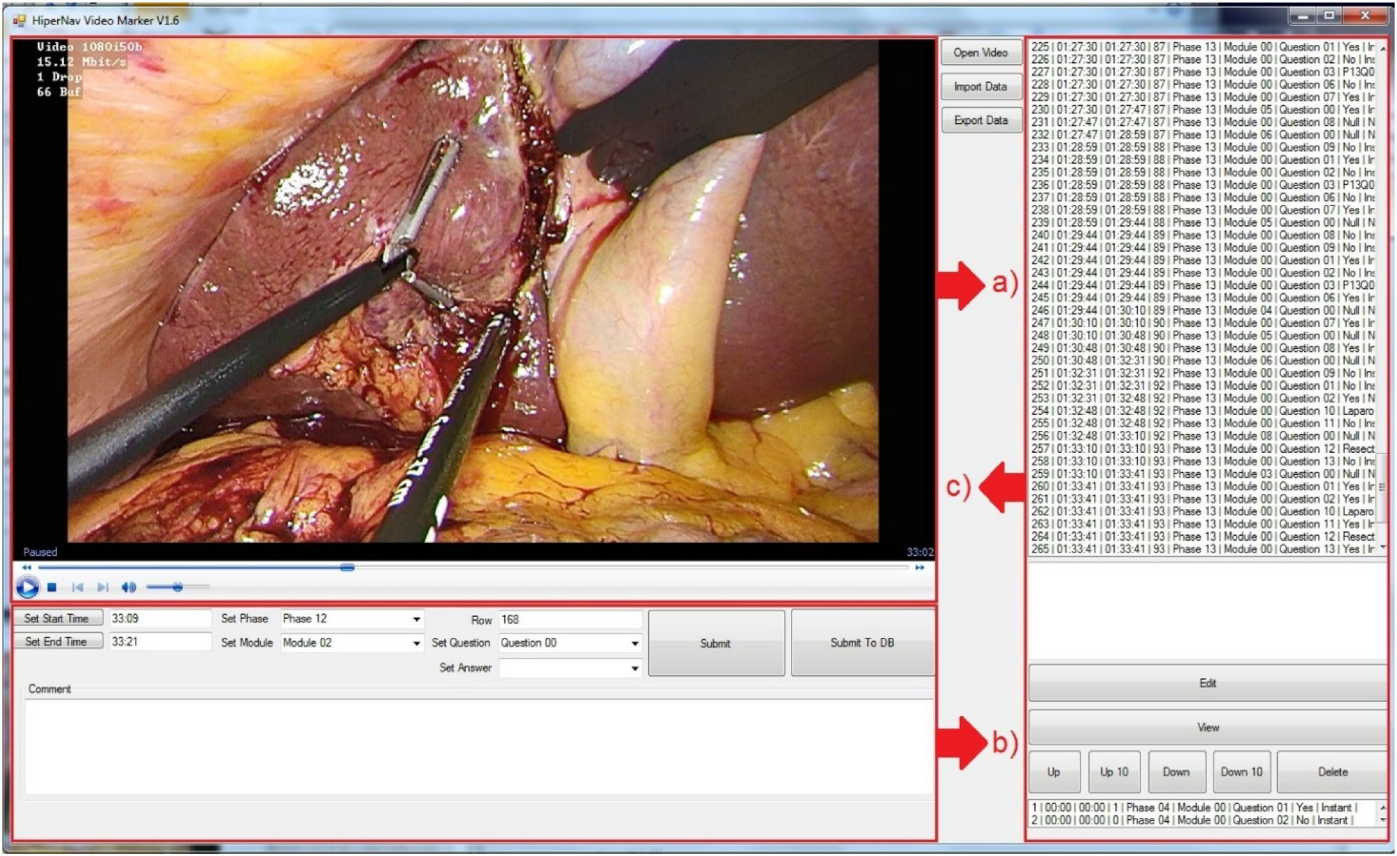
A snapshot of the developed process model data registration software (DOI: 10.4121/20163926). The software comprises three main sections: (**a**) endoscopic video player, (**b**) data registration panel to register data at the desired granularity level, locally or in the data-base and (**c**) registered data management.

As each treatment procedure can be composed of thousands of steps, in-house process model data registration software was developed to facilitate registration of data on the videos of the endoscopic camera (Fig. 2).

## Results

### Workflow phases

Within the MILT treatment procedure, including its preparations, three hierarchical sub-phases are distinguished clinically:

- **Operation:** the entire process **in the OR**, from when OR and patient are being prepared, until when the patient is moved out of the OR to the recovery room.
- **Intervention:** starts with the first ablation needle manipulation or first incision in the abdomen by the interventionist/surgeon and ends when the last incision is closed.
- **Surgery:** starts with the first incision in the abdomen by the surgeon and ends when the last incision has been closed.
- **Treatment:** the actual physical treatment (resection or ablation) of the target region.

**Figure 3:**
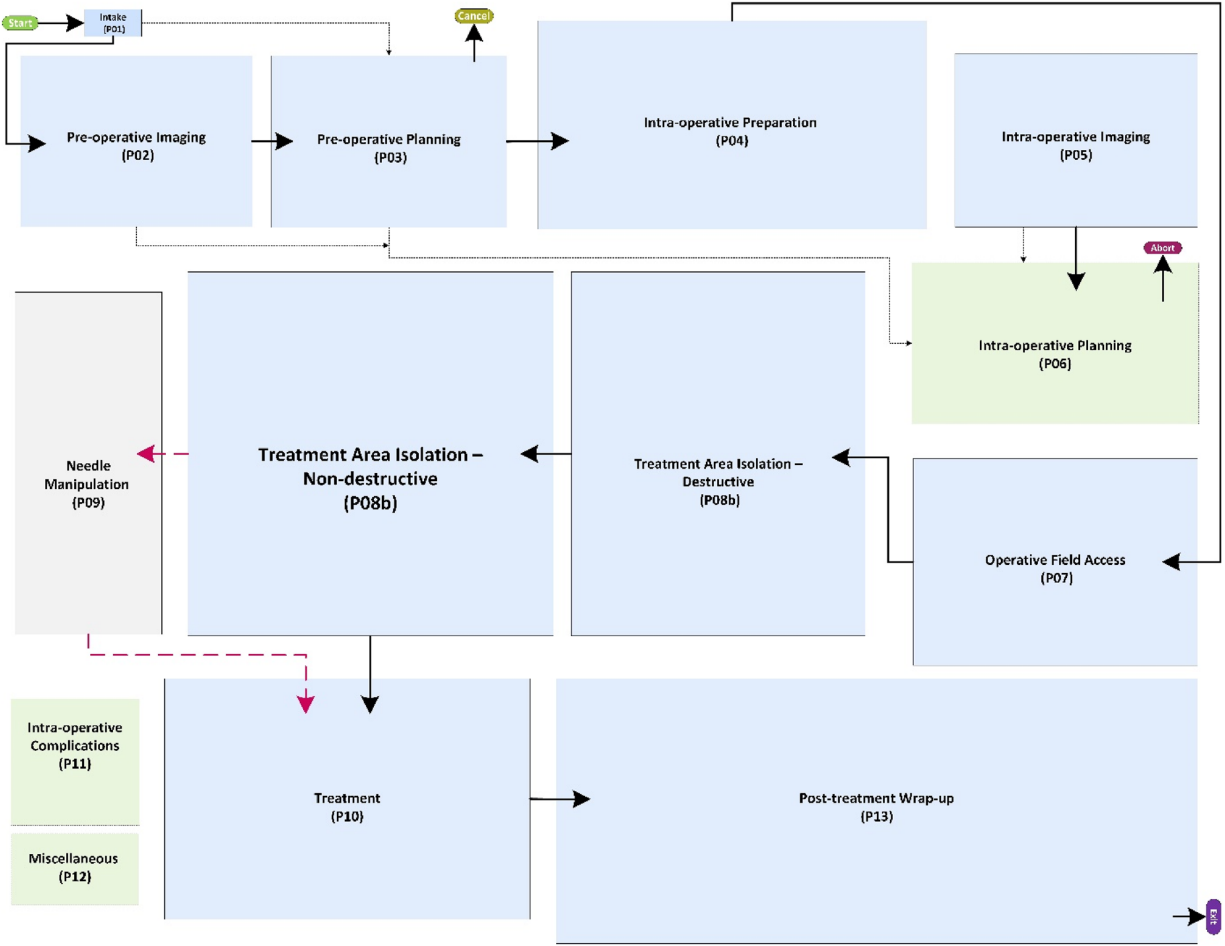
Generic process model of MILT at the phase level. Most of the phases are colored blue with solid-line rectangles; these phases are common between ablation and resection procedures. The gray phase, “needle manipulation”, is designated only for the ablation procedures. The blue and gray phases are connected by black solid and red dashed arrows showing the flow of activities. The black solid arrows are common between ablation and resection procedures, whereas the red dashed arrows are only used for ablation procedures. The green dashed rectangles show the phases that can happen anytime during the operation. These phases are connected to all other phases, but for the sake of readability, these arrows were left out of the figure. The black dotted-dashed arrows show the transfer of data such as medical images and patient medical history.

The generic process model of MILT procedures at the lowest granularity level (highest abstraction) is displayed in Fig. 3, showing all phases. The individual phases are explained below:

**Phase 01: Intake**—The patient is admitted to the hospital and complete anamnesis is collected.

**Phase 02: re-operative Imaging**—Medical images of the abdominal region are made for planning the MILT procedure prior to a possible operation. Phase 02 can take place right before operation up to a few months prior.

**Phase 03: Pre-operative Planning**—Planning includes all decisions about things like treatment approach, incision locations and resection paths or possible needle placements, size of the target region, etc. prior to the possible operation. The planning is based on the patient anamnesis (from Phase 01), medical images (from Phase 02), available equipment and technical resources and experiences.

**Phase 04: Intra-operative Preparation**—On the day of operation, prior to the intervention, the patient, OR equipment and surgical instruments are prepared for the operation.

**Phase 05: Intra-operative Imaging**—Medical images can be acquired in the OR, before and during the intervention.

**Phase 06: Intra-operative Planning**—The treatment plan can be generated or updated in the OR just before and during the intervention. Any pre-operative data and new images were taken in the OR (from Phase 05) aid in making decisions in this phase. The MILT *method* and *type* might also be changed during the operation. The MILT procedure is considered aborted if it is converted to a non-MILT procedure, such as open surgery.

**Phase 07: Operative field Access**—If LLR or LLA is the preferred method, the surgeon first makes the operative field accessible.

**Phase 08a/b:** Isolation of the treatment area consists of activities to separate the target region from surrounding structures and prepares the target region for the treatment. Based on the nature of these activities and how they affect the patient’s anatomy, isolation can be performed in two ways:

**Phase 08a: Treatment Area Isolation: Destructive**—Isolation by destructive (permanent) dissection or closure of surrounding structures. Only applies to LLR and LLA.

**Phase 08b: Treatment Area Isolation: Non-destructive**—Isolation with temporary effects, using actions such as temporarily closure of vessels or hydro dissection.

**Phase 09: Needle Manipulation**—Maneuvering ablation needle(s) to the desired position.

**Phase 10: Treatment**—The actual treatment of the target region by either resection or ablation.

**Phase 11: Intra-operative Complications**—Handling any complications that might occur during the operation. Such actions may include, for example, blood transfusion and hemostasis (e.g. bleeding vessel ligation) or surgical drainage.

**Phase 12: Miscellaneous**—Other clinical activities that do not directly serve the MILT procedure might take place, such as biopsy and catheter placement.

**Phase 13: Intra-operative Wrap-up**—All activities aimed at wrapping up, such as removal of un-absorbable materials, closing the incisions, etc.

**Figure 4:**
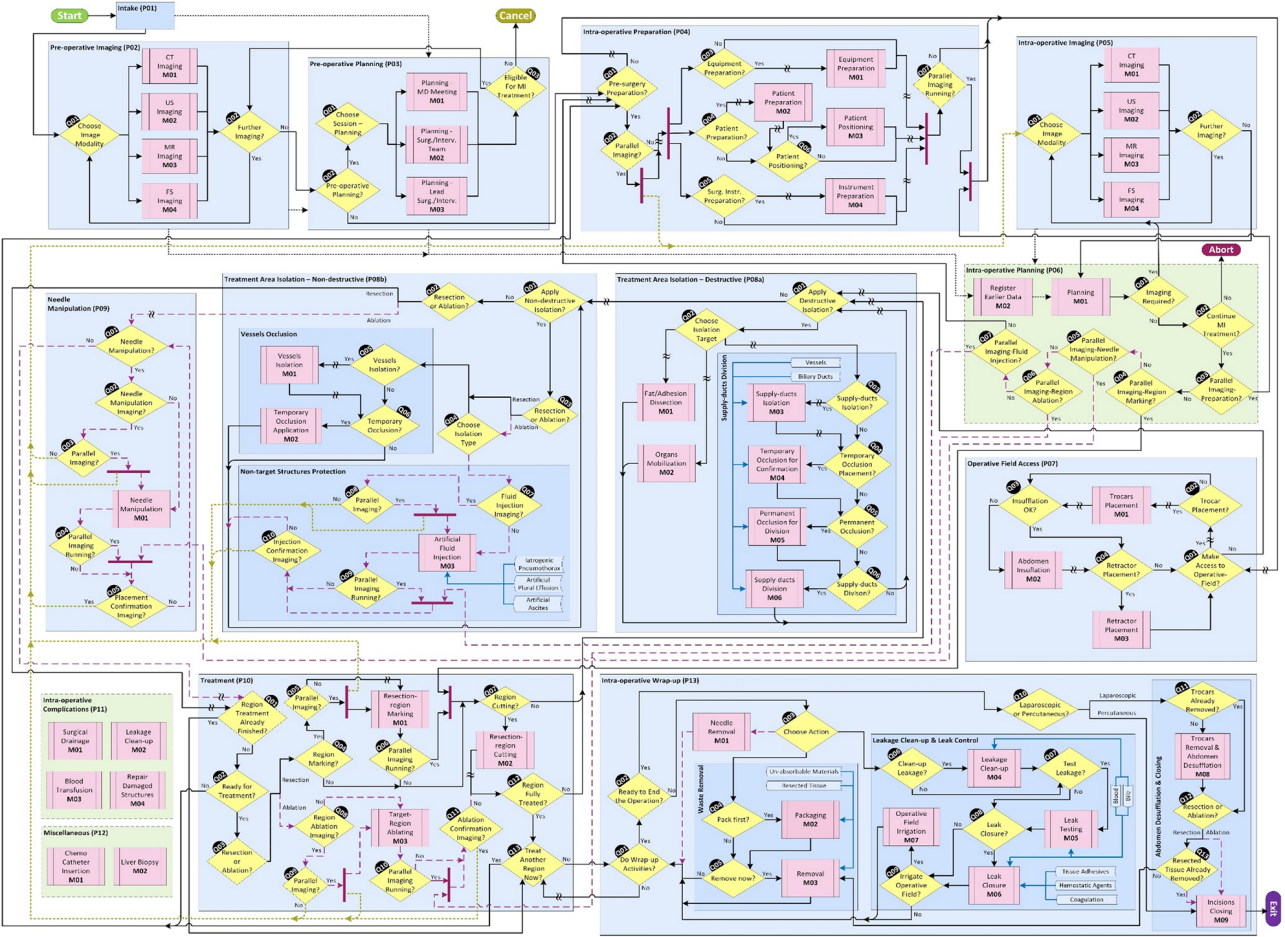
Generic process model for MILT procedures at the module granularity level. See Fig. 5 for explanation of the used symbols and line styles. DOI: 10.4121/20163968.

**Figure 5:**
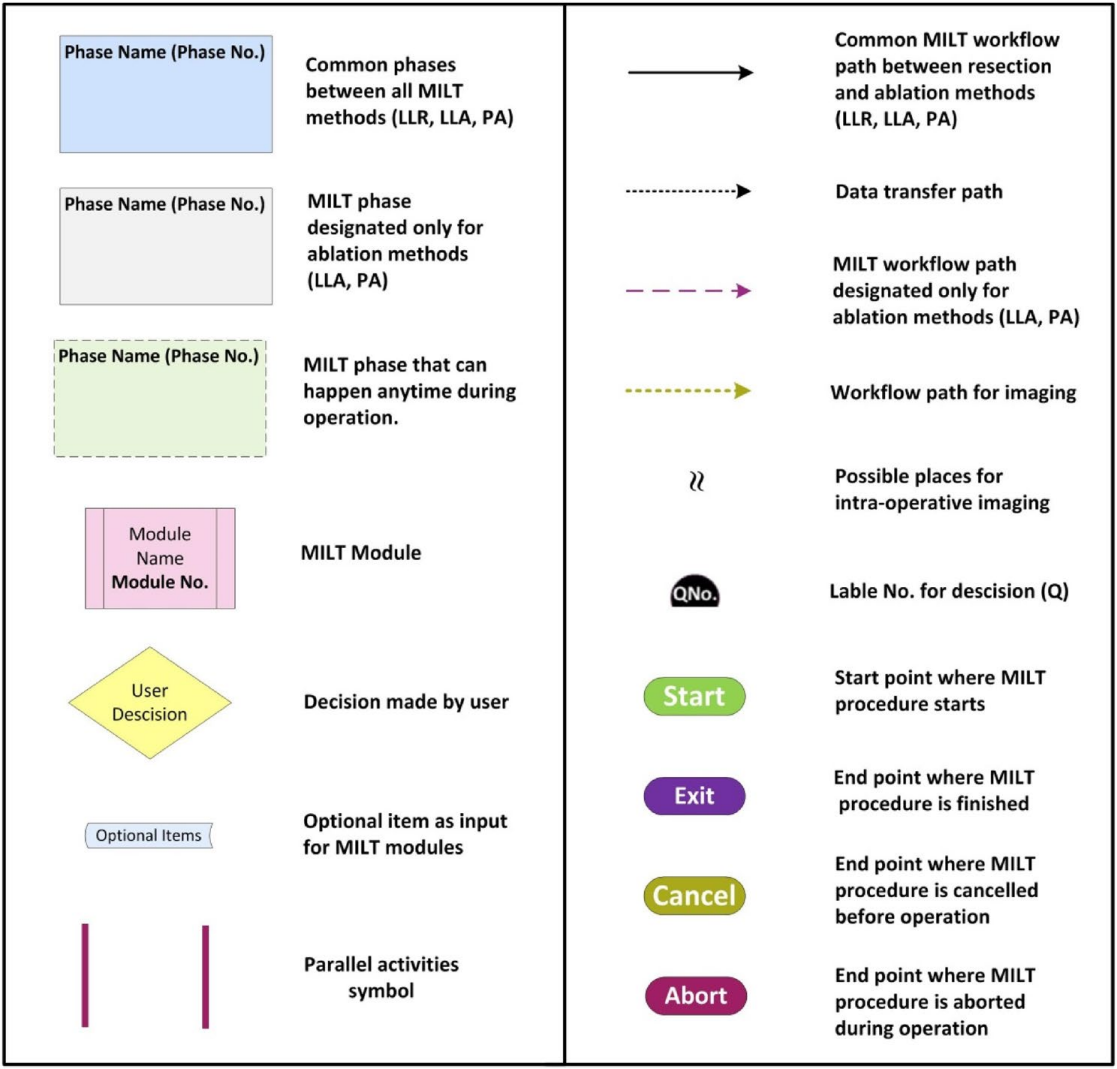
Explanation of the symbols and arrow styles used in Figs. 3 and 4.

The generic process model of MILT procedures at the module level, including the phases, modules and decisions linking the modules, is provided in Fig. 4 (DOI:10.4121/20163968). A legend explaining the different symbols used in Figs. 3 and 4 is provided in Fig. 5. All activities in the entire procedure of MILT including sequential and parallel activities are covered in the presented generic process model. Parallel activities are represented using  symbols. Apart from the continuous support of nurses and anaesthesiologist in the entire intra-operative phases and the act of blood suctioning in parallel to other treatment activities during surgery, based on the current data, the parallel activities are associated with two phases: intra-operative preparation phase (Phase 4) and intra-operative imaging (Phase 2) activities. In intra-operative phases, we plotted the connections associated with the imaging phase, where there was a high chance of performing imaging routines. In other places where this is less likely to happen, we used a symbol  to show the possibility for imaging. A brief walkthrough of the module-level MILT process model including the contents of the modules in the process model is provided in Supplementary material-part S1. A brief description of the Modules is provided in Table 1.

**Table 1:** Different phases of generic process model of MILT and the corresponding modules according to Fig. 4.

Phase	Modules	Description
Intake (01)	–	All relevant patient information is gathered
Pre-operative imaging (02)	CT imaging (1)	Different type of imaging modalities that provide different level of information of patient internal structures prior to the operation
	US imaging (2	
	MR imaging (3)	
	FS imaging (4)	
Pre-operative planning (03)	MD meeting (1)	Different planning meetings with different purposes can be carried out before the operation MD meeting (M01), so-called multidisciplinary team meeting to decide on the treatment approach. Surgical/interventional team meeting (M02) to discuss the equipment/instrument/patient preparation. The lead surgeon/interventionist (M03) session to pre-visualizes the whole procedure and all its key steps
	Surg./interv. team meeting (2)	
	Lead surg./interv. meeting (3)	
Intra-operative preparation (04)	Equipment preparation (1)	Preparations need to be carried out before the starts of the operation The equipment (M01), patient (M02) and instruments (M04) are prepared and the patient is positioned (M03) based on the pre-operative plan. These four modules are usually executed in parallel
	Patient preparation (2)	
	Patient positioning (3)	
	Instrument preparation (4)	
Intra-operative imaging (05)	CT imaging (1)	Different types of imaging modalities that provide different levels of information during the operation
	US imaging (2)	
	MR imaging (3)	
	FS imaging (4)	
Intra-operative planning (06)	Planning (1)	In the Planning (M01) the clinician can use the intra-operative images and endoscopic video, as well as the data from M02, to generate/update plan according to patient’s current condition and anatomy in the OR In Register Earlier Data (M02) the data of the pre-operative planning and imaging are registered to be used for the intra-operative planning
	Register earlier data (2)	
Operative field access (07)	Trocar placement (1)	In laparoscopic methods (LLR, LLA) the surgeon makes the operative field accessible. Trocar placement (M01) and the patient’s abdomen insufflation (M01) with carbon dioxide are performed to obtain access to the operative field. The surgeon can also place a fixed retractor (M03) to hold the liver or its surrounding organs
	Abdomen insufflation (2)	
	Retractor placement (3)	
Destructive isolation (08a)	Fat/adhesion dissection (1)	This phase includes three main actions: fat/adhesion dissection (M01), mobilization of the liver or its surrounding organs (M02) or dividing the supply ducts (M03, M04, M05 and M06). In order to safely divide the supply ducts, the surgeon might need to first isolate the ducts (M03) from their surrounding tissues and structures. Prior to the division of the supply ducts, they are occluded (M05) with care. Temporary occlusion of supply ducts (M04) might be required in order to confirm the location and closure of the target vessels (usually in formal/major resection). After the supply ducts are confirmed and occluded, they can be divided (M06)
	Organ mobilization (2)	
	Supply ducts isolation (3)	
	Temporary occlusion for division (4)	
	Permanent occlusion for division (5)	
	Supply ducts division (6)	
Treatment area isolation—non-destructive (08b)	Vessels isolation (1)	This phase includes two categories of actions. In case of laparoscopic procedures (LLR and LLA), the surgeon can first isolate any relevant vessels (M01) and then occlude them temporarily (M02) in order to reduce bleeding during treatment of the target region (e.g. Pringle maneuver). In case of ablation methods (LLA and PA), the surgeon/interventionists can inject buffer media (M03) between a lesion and the non-target nearby anatomical structures to protect them by absorbing extra energy
	Temporary occlusion application (2)	
	Artificial fluid injection (3)	
Needle manipulation (09)	Needle manipulation (1)	In the case of ablation, one or several needles are inserted through the skin to be placed at the desired position (M01) under the guidance of continuous or sequential medical imaging in the OR either. New images are also normally taken to confirm the needles are placed at the desired position
Treatment (10)	Region marking (1)	In the case of LLR, the surgeon needs to determine the resection margins and might need to mark (M01) physically on the organ (common in case of parenchyma sparing resection). The surgeon can proceed with cutting the resection region (M02). In the case of LLA and PA new images are normally needed before and/or during ablation (M03)
	Resection region treatment (2)	
	Target region ablation (3)	
Intra-operative complications (11)	Surgical drainage (1)	Complications might arise during the operation. In order to cope with these complications, different actions may have to be initiated, e.g. placing surgical drainage (M01), blood transfusion (M02), repairing damaged structures (M04) and cleaning up leakage (M03) from damaged structures
	Leakage clean-up (2)	
	Blood transfusion (3)	
	Repair damaged structures (4)	
Miscellaneous (12)	Chemo catheter insertion (1)	During the operation, various activities might be carried out that do not directly serve MILT e.g. inserting a catheter into a vessel (M01) to deliver chemotherapy medications or performing a liver biopsy (M02) for further examinations
	Liver biopsy (2)	
Wrap-up (13)	Needle removal (1)	After the treatment, the surgeon/interventionist tidies up and closes the operative field: ablation needle removal (M01), waste removal (M02 and M03), leakage clean-up and leak control (M04, M05, M06 and M07), and abdomen desufflation and incision closing (M08 and M09)
	Packaging (2)	
	Removal (3)	
	Leakage clean-up (4)	
	Leak testing (5)	
	Leak closure (6)	
	Operative field irrigation (7)	
	Trocars removal and abdomen desufflation (8)	
	Incisions closing (9)	

### Model verification

**Figure 6:**
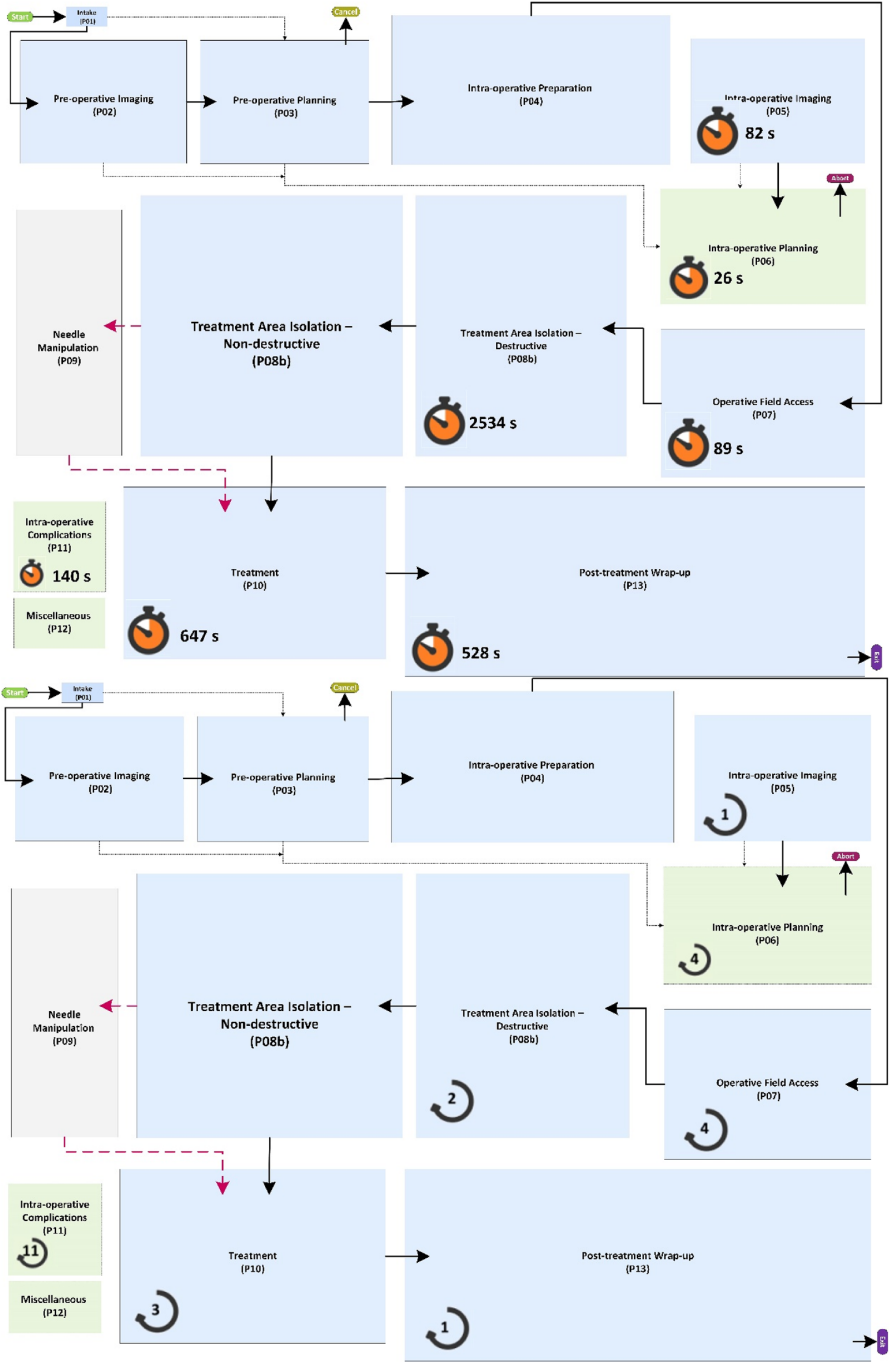
Generic surgical process model view at the phase level for duration and occurrence frequency of different phases for a sample surgery presented in Supplementary material-part S2 (type: parenchyma sparing of a tumor in Segments 5 and 6).

The result of the quantitative and qualitative verifications of the process model confirmed that the process model provides a pathway for all encountered sequences of actions and decisions that were observed to occur during MILT procedures in clinical practice. Supplementary material-part S2 lists all the registered sequence of actions and decisions in the entire duration of endoscopic videos from different surgical procedures for parenchyma sparing of a tumor located in Segments 5&6, 7&8 and 5, performed in OUH. Durations of all entities in the procedure are presented in the Supplementary material-part S2. Table 2 shows the duration and occurrence frequencies of every action extracted from the endoscopic video on which the Supplementary material-part S2 data is based, at the module as well as the phase granularity level. Figure 6 provides a process model view at the phase level for duration and occurrence frequency of different phases for the typical example of a surgical procedure. Note that during the entire course of a surgery, some timings are out of the view of endoscopic camera or associated with activities other than surgical actions, e.g. the surgeon might need to take out the camera and clean it. The timing of such activities are also extracted and labeled as Idle. Phases 1 to 3 are pre-operative phases and are not captured by the endoscopic videos. These pre-operative phases were verified through attendance to pre-operative imaging and planning sessions and discussions with clinical teams. The result of the verification process shows that there were no activities in any of the observed MILT procedures that were not covered by the proposed process model.

**Table 2:** The results of analysis on the data extracted from the endoscopic video in the both granularity levels of module and phase for a sample surgery (type: parenchyma sparing of a tumor in Segments 5 and 6 presented in Supplementary material-part S2.

Phase name (number)	Phase		Module name (number)	Module	
Phase	Duration (s)	Occurrence	Module	Duration (s)	Occurrence
Imaging (05)	82	1	Imaging (2)	82	1
Planning (06)	26	4	Planning (1)	26	4
Operative field access (07)	89	4	Trocar placement (1)	89	4
			Abdomen insufflation (2)	0	0
Destructive isolation (08a)	2534	2	Fat/adhesion dissection (1)	90	1
			Organ mobilization (2)	518	1
			Supply ducts isolation (3)	842	21
			Temporary occlusion for division (4)	0	0
			Permanent occlusion for division (5)	267	5
			Supply ducts division for (6)	817	20
Treatment (10)	647	3	Region marking (1)	171	1
			Resection region treatment (2)	476	2
Intra-operative complications (11)	140	11	Leakage clean-up (2)	140	11
Wrap-up (13)	528	1	Packaging (2)	76	1
			Removal (3)	121	1
			Leakage clean-up (4)	112	3
			Leak testing (5)	0	0
			Leak closure (6)	219	4
			Operative field irrigation (7)	0	0
Idle				157	
			Sum	4203

In sessions with two highly experienced surgeons and two assistant surgeons in OUH and Erasmus MC, discussing the validity and correctness of the generic process model for different techniques of performing MILT, it was confirmed that the proposed process model mimics the activities in the clinical practice.

## Discussion

Surgical process models bring several advantages and pave the way for further improvement of operations. The presented generic process model covers a broad range of MILT procedures and associated techniques. No deviations from the proposed process model were found in the treatment procedures that were analysed in the verification process. The proposed process model provides relationships between different entities of MILT procedures at the proposed levels of details. Thus, the process model provides the possibility for extensive quantitative as well as qualitative analysis of the procedures at the desired level of detail.

In intra-operative phases, distinguishing between planning and other treatment activities is a complicated task. Planning during operation is an ongoing mental activity and can be considered as an element inside all intra-operative phases. Modelling planning activities in a separate phase in the generic process model provides the foundation for further analysis and improvement of planning. Recognizing the points where planning occurs in the surgical process model and deriving the sequential relationships between planning and other intra-operative activities, show how and to what extent planning is associated with different activities and reveals the possible bottlenecks of planning.

Imaging activities can occur at any moment in the intraoperative phases. Although imaging activities could be defined as a green phase in the proposed generic surgical process model, it was decided to model sequential and parallel dependencies between entities as it highly benefits further analysis of process model and performing possible simulations. Live observations and interviews with experts in two institutions (OUH and Erasmus MC) were performed to determine the low granularity level structure of the process model. The process model was initially established based on the data from endoscopic video analysis and live observations in aforementioned institutions. The data was complimented with literature studies and analysis of videos of procedures available on the web from different institutes (Institute of Medical Education of Novgorod State University in Russia and Unité Hepatobiliopancreatique in Strasbourg, France- Videos can be found at Dr Sergey Baydo (https://www.youtube.com/c/DrSergeyBaydo/videos) and Dr Riccardo Memeo (https://www.youtube.com/channel/UCdhB0tuE3EC_iNipn1A3ltg/videos) YouTube Channels.) to make the process model as generally applicable as possible. Moreover, in verification process, the endoscopic videos of fifteen additional surgeries performed in OUH were analysed and six live observations of MILT procedures were performed in Erasmus MC and BUH. For these reasons, process model should conform to the procedures in other institutions as well. In this study, we did not take videos from the OR. These recording would make further quantitative validation of the model possible, but also requires special ethical approval, since sensitive information is recorded. In an earlier study, we investigated the consequence of recording in the OR^66^. In this study the pre-operative and not the post-operative phase was included in the generic process model, because the former has a direct influence on performing the treatment, which is the focus of this work. All concepts associated with different techniques of MILT are defined and categorized as different phases and modules. Thus, we expect that variations of performing actions in different institutions by using different techniques/instrument, will hardly cause any deviations from the proposed process model. However, lack of instruments, equipment or knowledge might change the course of actions or introduce innovative ways to tackle problems (that might happen especially in underdeveloped countries), which may not be considered in the presented process model. Recognizing and registration of surgical activities are crucial for performing analysis on surgical procedures, generating and verifying surgical process models and training machine learning methods to develop AI systems for the future hybrid ORs^67^. The in-house developed Video Marker Software in this work aided efficient registration and verification of data over the endoscopic video. The extracted data using the Video Marker Software from surgical videos that are acquired from OUH has been presented in Supplementary material-part S2. The statistical analysis of the extracted data reveals the bottlenecks in different surgeries. Based on the analysis, the surgeons spent most of their time on the treatment phase (P10); approximately 25 min (40% of total surgery time), and almost 85% of the treatment phase duration was allocated to the resection. This result emphasises the importance of treatment phase on the total surgery duration. Development of automated workflow recognition systems that can (semi)automatically analyse the endoscopic videos with appropriate image processing and/or machine learning methods are currently under attention of researchers, especially for analysis of minimally invasive treatments^68,69^. Such systems can be of great use to aid gathering surgical data for different purposes of process model analysis and verifications^67,70,71^.

The presented process model aids different aims of analysis for improvement of surgeries/interventions in follow-up studies. Analysis of process models and providing connections between every entities of the surgical procedures, identify the points where AI and software/platform systems can be beneficial, predicts how big the benefits are and determines how these systems can be designed and developed to be employed in clinical practice, see e.g. Ref.^4,72^. Development phase of the desired technologies and tools for hybrid ORs can also benefit from analysis of such surgical process models. Nowadays, Agile methods (SCRUM, XP, etc.)^73,74^ are being widely used in the process of the development of technologies. These methods aid smooth adaptation to changing requirements throughout the development process by using iterative planning and feedbacks from developers and the end users^73,75^. With the process model and computer simulations, analysis of the effect of possible changes and their eligibility aids making right decisions and adaptations during the agile sessions.

The process model can widely contribute in the training and skill evaluation of surgeons^76–78^. The optimal treatment option for each surgery with specific conditions can be derived and novice surgeons can be trained based on the probable sequence of events and the possible deviations for each operation. The experienced surgeons can review the steps and possible deviations before or during an operation as a roadmap. For this purpose, real-time recognition of surgical steps over the endoscopic videos is required, a topic which has attracted wide attentions in recent years^79,80^. The process model benefits analysis of surgeons’ learning curves^81,82^. Durations and occurrence frequencies of surgical steps and deviations from nominal surgery paths can be used as criteria for learning curve analysis, as well as surgeons’ skills evaluations. In recent years navigation platforms for guiding surgeons in performing MILT attracted broad attention^83–88^. Analysis of the proposed surgical process model can reveal the optimal treatment options to guide surgical teams using navigation systems by suggesting/predicting next surgical steps and the time required for performing each surgical action^5,13,89,90^. Currently, prior to operation the lead surgeon/interventionist goes into the details of the patient’s organ-specific anatomy and mentally pre-visualizes the whole procedure and all its key steps. The complexities of such pre-operative planning activity, can be reduced by the process model which brings the possibility to propose the treatment options for individual procedures. Analysis of surgical process model can prevent extra costs of trial and error in the development phase of technologies and introduction of new technologies into clinical practice. With the process model, it is possible to provide scientific evidence for the possible enhancement of surgeries by the proposed technology for specific methods/types/techniques of performing surgeries. The effects and eligibility of any adjustment in the new technologies can be analysed on the surgical procedure, prior to actual implementation of technologies, resulting in a more efficient business model.

## Conclusion

A generic surgical process model for MILT was established by applying the modelling strategies developed in prior work. The presented model covers MILT methods for laparoscopic liver resection, laparoscopic liver ablation and percutaneous ablation, with their types, techniques and variations as observed in data obtained from various sources. As the presented model was established using a numerical model representation, it can be used for extensive quantitative and qualitative analysis and improvement of MILT procedures through various ways, such as the introduction of new technologies in the OR, training of clinical teams, analysis of learning curves and skills evaluations, optimization of OR management and medical team activities in the OR.

## Supplementary Information

Supplementary Information.

## Data Availability

All data generated or analysed during this study are included in this published article and its supplementary information files. The datasets generated and/or analysed during the current study are also available in the DOI: 10.4121/20163968.
