# Supplementary material for: Generic surgical process model for minimally invasive liver treatment methods
Source: Sci Rep. 2022 Oct 6;12:16684. doi: 10.1038/s41598-022-19891-1 (PMC9537522; doi:10.1038/s41598-022-19891-1)
Supplement: Supplementary file 1 — Supplementary Information. [file 41598_2022_19891_MOESM1_ESM.docx]

**GENERIC SURGICAL PROCESS MODEL FOR MINIMALLY INVASIVE LIVER TREATMENT METHODS**

Maryam Gholinejad^1*^, Egidius Pelanis^2,3^, Davit Aghayan^2,4^, Åsmund Avdem Fretland^2,5^, Bjørn Edwin^2,3,5^, Turkan Terkivatan^6^, Ole Jakob Elle^2^, Arjo Loeve^1^, Jenny Dankelman^1^

^1^ Department of Biomechanical Engineering, Faculty of Mechanical, Maritime and Materials Engineering , Delft University of Technology, Delft, the Netherlands.

^2^ The Intervention Centre, Oslo University Hospital, Oslo, Norway

^3^Institute of Clinical Medicine, Medical Faculty, University of Oslo, Norway.

^4^Department of Surgery N1, Yerevan State Medical University after M. Heratsi, Yerevan, Armenia.

^5^Department of HPB Surgery, Oslo University Hospital, Oslo, Norway.

^6^ Department of Surgery, Division of HPB & Transplant Surgery, Erasmus MC, University Medical Centre Rotterdam, Rotterdam, the Netherlands.

## APPENDIX 1 - Workflow modules

In this appendix, the MILT generic process model at a module level, shown in Figure 3, is explained by a brief walkthrough model and its modules. Modules inside the phases are annotated by an ‘M’ proceeded by the module number.

**Phase 01:** **Intake** - All relevant patient information is gathered.

**Phase 02:** **Pre-operative Imaging** - Images are taken prior to the operation, using any preferred image modalities. Different imaging modalities (M01, M02, M03 and M04) provide different levels of information related to internal organs, bones, soft tissues, or blood vessels.

**Phase 03:** **Pre-operative Planning** - Planning sessions are held prior to the operation and involve a wide variety of clinical personnel. Typically, there is a planning session (M01), so-called multidisciplinary team (MDT) meeting, during which the clinical experts (surgeons, interventionists, radiologists, and gastrointestinal experts, etc.) discuss the patient’s condition and decide on the treatment approach. If MILT is chosen as the treatment approach, before the operation there can be a different preparation/planning sessions between the surgical/interventional team members (M02) (typically lead surgeon/interventionist, surgeon/interventionist assistant and head nurse) to discuss the patient preparation, required instruments and equipment, and any required deviations from standard protocols. Finally, the lead surgeon/interventionist (M03) goes into the details of the patient’s organ-specific anatomy and mentally pre-visualizes the whole procedure and all its key steps. In the planning sessions, the clinicians may require new or additional images for different reasons, such as higher quality or extra or updated information. Note that not all meetings are always held and that any can be repeated if necessary. The planning contains any decisions about the desired treatment method, type and techniques, preparation methods and required instruments and equipment, adequate margin for the target region, optimal path to reach the target region, etc.

**Phase 04:** **Intra-operative Preparation** - Before the surgeon/interventionist starts the treatment procedure, all required equipment is placed in the OR (M01). The sterile nurse together with another nurse(s) prepare the surgical instruments (M04), the patient (M02) and position the patient according to the plan (M03). These four modules are usually executed in parallel. Acquiring new images of the patient’s organ is also possible in parallel to performing preparations in this phase.

**Phase 05: Intra-operative Imaging** – Intra-operative imaging can be initiated from numerous places in the workflow during the operation. Therefore, to avoid cluttering the process model scheme, the
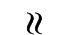
symbol is used to indicate jump-outs to possible imaging. If it is highly probable or standard procedure to call imaging modalities at any point, the corresponding decisions and arrows are plotted with thick green dotted-dashed lines. After new images are acquired (M01, M02, M03 and M04), the surgeon/interventionist always checks if an update of the treatment plan is needed.

**Phase 06: Intra-operative Planning** – Any aspects of the treatment plan can be generated or updated in the OR. The process model is flexible to apply these changes. Therefore, the next step after the pre-operative planning is always Phase 04, where **operation** starts. The clinician can use intra-operative images and endoscopic video to generate/update plan according to patient’s current condition and anatomy in the OR.

**Phase 07: Operative Field Access** - In laparoscopic methods (LLR, LLA) the surgeon makes the operative field accessible. Firstly, the first trocar is inserted into the patient’s abdomen (M01). Typically, when the insertion of the first trocar is successful, the abdomen is insufflated with carbon dioxide (M02). The surgeon continues inserting more trocars according to the plan and need for sufficient insight and tool access. In case of improper abdominal insufflation, the surgeon decides whether to manipulate the trocar in its current insertion point or to change the location of the first trocar. In hand-assisted laparoscopic surgery [79] the surgeons can use their hand instead of a trocar through a hand port. The surgeon can also place a fixed retractor (M03) to hold the liver or its surrounding organs throughout the surgery, whenever needed after successful insufflation.

**Phase 08a: Treatment Area Isolation – Destructive** - In this phase, the surgeon can choose between three main actions: fat/adhesion dissection (M01), mobilization of the liver or its surrounding organs (M02) or dividing the supply ducts (M03, M04, M05 and M06). Typically, the surgeon dissects fat/adhesion (M01) for different reasons: to reach the treatment area, to have a better view of the treatment region, etc. Mobilization of the liver (M02) involves dissection of ligamentous/peritoneal attachments and if present any adhesions. Small branches of supply ducts can be easily occluded and divided using coagulation devices, while the division of larger branches of supply ducts require special care. The surgeon might need to close and divide supply ducts for different reasons such as blocking the fluid exchange between the treatment area and healthy parenchyma (e.g. in case of formal resection), and while performing the treatment (e.g. in case of PR). In order to safely divide the supply ducts, the surgeon might need to first isolate the ducts (M03) from their surrounding tissues and structures. Prior to the division of the supply ducts, they are occluded (M05) with care. As in MILT procedures, the field and quality of view are limited, confirmation (M04) of the location and closure of the target vessels with other techniques is sometimes required before performing permanent occlusions. To this purpose, the surgeon can temporarily occlude any supply ducts and observe the effect of blocking the blood supply to the target tissue (usually in formal/major resection). After the supply ducts are confirmed to be occluded, they can be divided (M06). Several devices are available that allow to occlude and divide the ducts in a single action (e.g. Stapler). Note that in the case of parenchyma sparing and anatomical resection, the activities in supply duct division, can be considered in the treatment phase. However, for the sake of clarity and generality in the generic surgical process model, the activities in supply duct divisions are modelled in destructive isolation phase.

**Phase 08b: Treatment Area Isolation – Non-destructive** - The techniques in non-destructive isolation contribute to reduction of operative bleeding and promote better hemostasis while performing treatment of the target region, or protection of nearby structures. Two different categories of actions are available. In case of laparoscopic procedures (LLR and LLA), non-destructive isolation involves techniques for temporary vessels occlusion (M01 and M02), in which the surgeon first isolates any relevant vessels (M01) and then occludes them temporarily (M02) in order to reduce bleeding during treatment of the target region (e.g. Pringle maneuver). In case of ablation methods (LLA and PA), the surgeon/interventionists can inject buffer media (different types of liquids or gas [80]) (M03) between a lesion and the non-target nearby anatomical structures to protect them by absorbing extra energy. In such cases, the surgeon/interventionist uses medical imaging in the OR to guide the injection of buffer media. Similar approach could also be applied as direct cooling of the sensitive structures (e.g. bile duct cooling) (PMID: 15110804)

**Phase 09: Needle Manipulation** – In the case of ablation, one or several needles are inserted through the skin to be placed at the desired position. The interventionist places the needle(s) at the right position under the guidance of continuous or sequential medical imaging in the OR either. New images are also normally taken after needle manipulation to confirm the needles are placed at the desired position.

**Phase 10: Treatment** – Treatment of the target region can be done either by resection or ablation. In the case of LLR, the surgeon needs to determine the margins to apply when removing the targeted tissue volume. To do so, one might mark (M01) the resection region physically on the organ by using a coagulation device (common in case of parenchyma sparing resection). New images might be needed before and/or during marking. Once resection marking (partially) is completed, the surgeon can decide to proceed with cutting the resection region (M02). In the case of LLA and PA new images are normally needed before and/or during ablation. Continuous or sequential imaging during ablation are used to monitor and control the treatment progress. After a completed ablation, new imaging is preferably taken to check for any complication and to assess the ablative margin to better decide whether to proceed with the treatment or not. In all methods (LLR, LLA, PA) (non-)destructive isolation techniques can be applied while treating the target region, leading the workflow to the corresponding phases during resection or ablation.

**Phase 11: Intra-operative** **Complications** - Complications might arise during the operation. In order to cope with these complications, different actions may have to be initiated, e.g. placing surgical drainage (M01), blood transfusion (M02), repairing damaged structures (M04) and cleaning up leakage (M03) from damaged structures.

**Phase 12: Miscellaneous** - During the operation, various activities might be carried out that do not directly serve MILT. Inserting a catheter into a vessel (M01) to deliver chemotherapy medications after the operation or performing a liver biopsy (M02) for further examinations are two examples of these activities.

**Phase 13:** **Intra-operative Wrap-up**- After the treatment is finished, the surgeon/interventionist tidies up and closes the operative field by applying: ablation needle removal (M01), waste removal (M02 and M03), leakage clean-up and leak control (M04, M05, M06 and M07), and abdomen desufflation and incision closing (M08 and M09). The surgeon/interventionist often intermediately applies some of the wrap-up activities (M01 to M06) during surgery after having finished treating one or a part of one target region before proceeding to the next.

## APPENDIX 2 - Verification data:

In the verification process of the generic surgical process model of MILT in this work, the endoscopic videos were analyzed to acquire the order and duration of steps in the entire surgical procedures. The presented figures in this Appendix are the data extracted from the endoscopic videos of parenchyma sparing of tumor at segments 5&6, 7&8 and 5. In the following figures, the horizontal axis shows the different surgeries and vertical axis is the number of steps and. For each surgery the videos were analysed The numbers on the graphs are **P**hase number, **M**odule numbers and **D**uration of the step in seconds. Each phase has its own symbol and colour so these are easily distinguished. The Duration of the first few steps (trocar placement and its planning at the bottom of plot) are indicated as NaN at the start of each surgery as there is no record of these steps in the endoscopic videos. The steps in the surgeries are placed chronologically from bottom to top of the plots. The datasets generated during the current study are available in the DOI: [10.4121/20163968](https://urldefense.com/v3/__https:/doi.org/10.4121/20163968__;!!PAKc-5URQlI!6YXVizYb_yzBkrhGdJPQIRWD44iuPjlSmnZk1ptuW2Wv261H0acr9ZPLvRfd_814yn06Ezw2Ad-vQiBf8yNtYcUaT6R1aw$)

*
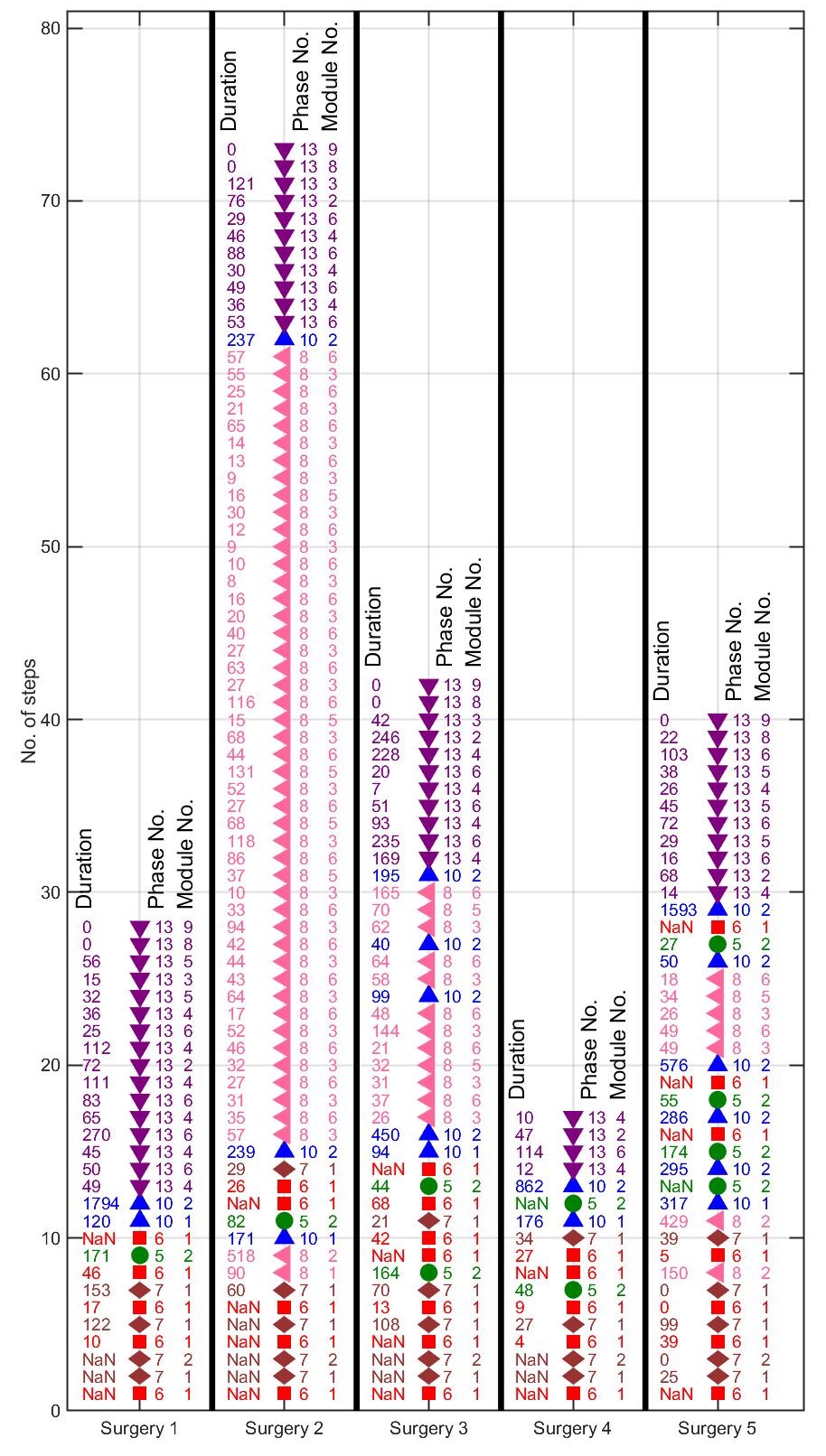
*

*Figure 1.B: The data for five endoscopic videos of laparoscopic liver resection of segments 5&6. Please see the main text above this figure for explanation of the figures and symbols.*


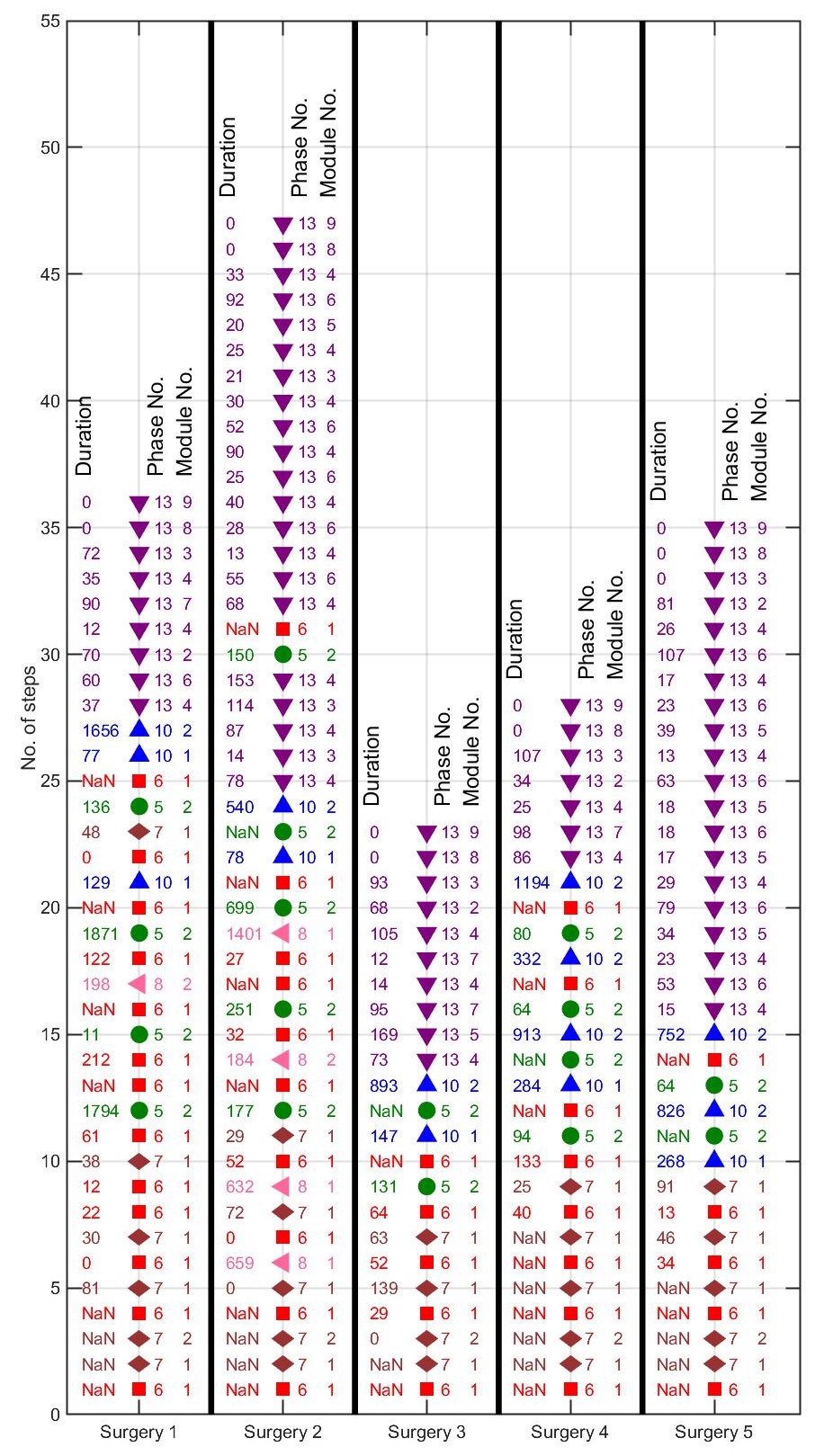


*Figure 2.B: The data for five endoscopic videos of laparoscopic liver resection of segments 7&8. Please see the main text above this figure for explanation of the figures and symbols.*

*
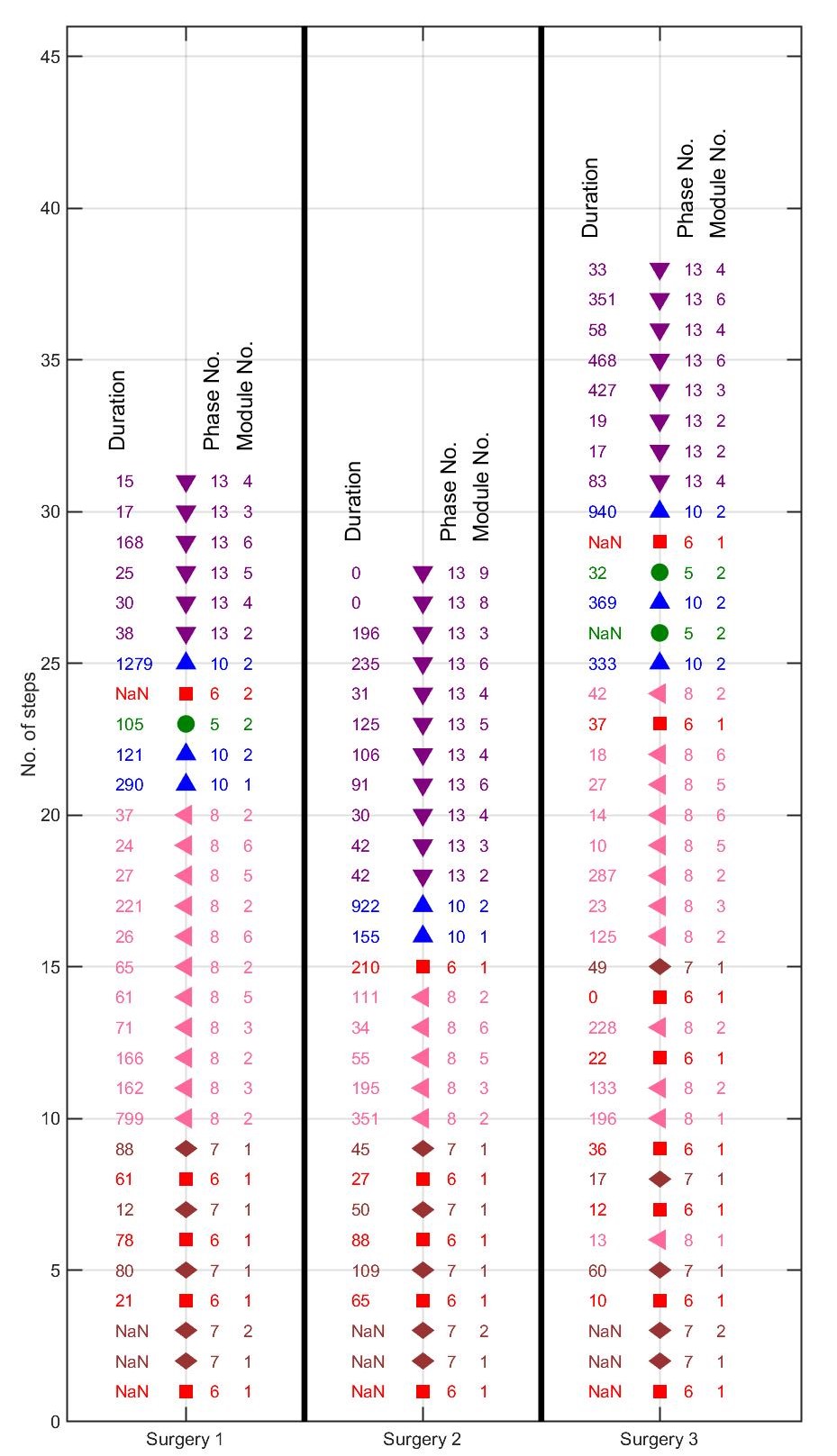
*

*Figure 3.B: The data for three endoscopic videos of laparoscopic liver resection of segments 5 with gallbladder removal. Please see the main text above this figure for explanation of the figures and symbols.*
